# Application of clotrimazole via a novel controlled release device provides potent retinal protection

**DOI:** 10.1007/s10856-015-5561-9

**Published:** 2015-09-03

**Authors:** Zhaleh Kashkouli Nezhad, Nobuhiro Nagai, Kotaro Yamamoto, Hirokazu Kaji, Matsuhiko Nishizawa, Hideyuki Saya, Toru Nakazawa, Toshiaki Abe

**Affiliations:** Division of Clinical Cell Therapy, United Centers for Advanced Research and Translational Medicine (ART), Tohoku University Graduate School of Medicine, 2-1 Seiryo-machi, Aoba-ku, Sendai, 980-8575 Japan; Department of Ophthalmology, Tohoku University Graduate School of Medicine, 1-1 Seiryo-machi, Aoba-ku, Sendai, 980-8574 Japan; Department of Bioengineering and Robotics, Graduate School of Engineering, Tohoku University, 6-6-01 Aramaki, Aoba-ku, Sendai, 980-8579 Japan; Division of Gene Regulation, Institute for Advanced Medical Research, School of Medicine, Keio University, 35 Shinanomachi, Shinjuku-ku, Tokyo, 160-8582 Japan

## Abstract

Age-related macular degeneration is the leading cause of legal blindness among older individuals. Therefore, the development of new therapeutic agents and optimum drug delivery systems for its treatment are crucial. In this study, we investigate whether clotrimazole (CLT) is capable of protecting retinal cells against oxidative-induced injury and the possible inhibitory effect of a sustained CLT-release device against light-induced retinal damage in rats. In vitro results indicated pretreatment of immortalized retinal pigment epithelium cells (RPE-J cells) with 10–50 µM CLT before exposure to oxygen/glucose deprivation conditions for 48 h decreased the extent of cell death, attenuated the percentage of reactive oxygen species-positive cells, and decreased the levels of cleaved caspase-3. The device consists of a separately fabricated reservoir, a CLT formulation, and a controlled release cover, which are made of poly(ethyleneglycol) dimethacrylate (PEGDM) and tri(ethyleneglycol) dimethacrylate (TEGDM). The release rate of CLT was successfully tuned by changing the ratio of PEGDM/TEGDM in the cover. In vivo results showed that use of a CLT-loaded device lessened the reduction of electroretinographic amplitudes after light exposure. These findings indicate that the application of a polymeric CLT-loaded device may be a promising method for the treatment of some retinal disorders.

## Introduction

Age-related macular degeneration (AMD) occurs primarily in elderly people and is the leading cause of legal blindness among older individuals in the developed world [1–3] It has been predicted that as the population ages, there will be a 50 % increase in the incidence of AMD before 2020 [4]. Pathophysiologically, AMD derives from pathologic alterations of retinal pigment epithelium (RPE) cells as well as its related tissues and their interactions with the local environment [5]. In particular, reactive oxygen species (ROS) generation by oxidative stress induced by ischemia and hypoxia in the related area, is considered to be a key factor in AMD pathophysiology [6]. The RPE is one of the major ocular tissues affected by oxidative stress and is known to play an important role in pathogenesis of AMD [7]. Therefore, the protection of RPE cells against oxidative damage may be important in retinal protection for the treatment of AMD [8].

The development of optimum drug delivery systems is of great importance as well as the discovery of new therapeutic agents [9]. At present, one of the delivery methods is topical application. However, potential treatments for posterior segment diseases using this approach are hampered by the barrier function of corneal epithelium and tear fluid turnover [10]. Additionally, the molecular size and physical characteristics of the substance affect its topical delivery [11]. Soluble substrates pass easily through the sclera because of its high degree of hydration and low cell population [12]. We reported that low-molecular compounds could reach the RPE via a transscleral route, accumulate around the RPE, and pass through the RPE into the neural retina [13]. Therefore, transscleral delivery is potentially a more applicable method for drug delivery to the posterior segment of the eye compared to topical application [14].

Drug repositioning (drug reprofiling, drug repurposing) is gaining importance as the development of new drugs becomes increasingly expensive [15]. Although only a few compounds have been approved for new indications in the field of retinal disorders, there are a number of substances with the potential to become reprofiled for new indications [16]. Clotrimazole, 1-(2-chloro-phenyl) diphenylmethyl1H-imidazole (CLT), is a potent antimycotic drug, acting via the inhibition of sterol-14-demethylase, a cytochrome P-450-dependent enzyme [17]. CLT is currently used in human and veterinary medicine for the treatment of fungal infections [18]. It has been suggested that it could also be effective for the treatment of malaria and tuberculosis [19, 20]. Furthermore, the neuroprotective effects of CLT have been proven previously [21]. Studies have also demonstrated the effects of this drug on ovarian ischemia/reperfusion injury and liver ischemia/reperfusion injury [22, 23].

The objectives of the present study were to investigate whether CLT could protect RPE cells against oxidation-induced injury and to examine the sustained release of CLT using a previously described polymeric device [13] with a goal of repositioning CLT as a drug for retinal disease treatment. Excessive light exposure leads to photoreceptor degeneration in many animals [24, 25] and can be a risk factor for the onset and/or progression of AMD [26]. In these pathological conditions, ROS generation is involved in cell death [27, 28]. Thus, preliminary implantation of the CLT-loaded device on the sclera of rats was performed to evaluate its retinal protective effect against light-induced retinal injury.

## Methods

### Materials

CLT, dimethylsulfoxide (DMSO), phosphate buffer saline (PBS) and penicillin (100 U/ml)/streptomycin (100 mg/ml) solution were purchased from Wako (Japan). Dulbecco’s modified Eagle’s medium (DMEM), fetal bovine serum (FBS) and l-glutamine were purchased from Gibco (Japan). A RPE cell line derived from primary cultures of RPE cells taken from 7-day-old Long-Evans rats (RPE-J cells) was purchased from ATCC (USA). Poly(ethyleneglycol) dimethacrylate (PEGDM, Mn 750), tri(ethyleneglycol) dimethacrylate (TEGDM, Mw 286.3) and 2-hydroxy-2-methylpropiophenone were purchased from Aldrich (USA). The reagents for high-performance liquid chromatography (HPLC) were purchased from KantoKagaku (Japan). Cell culture plates were purchased from BM equipment (Japan). A ProteoJET Cell Lysis kit was purchased from CosmoBio (Japan). SDS-PAGE reagents and electrophoresis gels were purchased from Biorad (Japan).

### In vitro cell culture

RPE-J cells were maintained in DMEM (45 mM glucose) containing 4 % FBS, 1 % penicillin/streptomycin solution and 4 mM l-glutamine at 33 °C in a 5 % CO_2_ humidified incubator. The cells were plated in 96-well culture plates at a density of 2 × 10^4^ cells/cm^2^ and incubated for 48 h. After culturing the cells in media (0.1 ml) containing CLT at predetermined concentrations (including 0.03 % DMSO as a solvent for CLT) for 24 h, the cells were exposed to hypoxic conditions (2 % oxygen) with no glucose (oxygen/glucose deprivation; OGD). The OGD exposure time was set to 48 h. Cell viability was assessed using a CellTiter 96 Aqueous One Solution Cell Proliferation Assay (Promega). 3-(4,5-dimethylthiazol-2-yl)-5-(3-carboxymethoxyphenyl)-2-(4-sulphophenyl)-2H-tetrazolium (MTS) solution mixed with medium in a ratio of 1:10 was applied to cells for 60 min and the MTS absorbance was measured at 492 nm (Fluoroscan Ascent).

### Measurement of intracellular ROS levels

To assess the effect of CLT treatment on ROS generation, RPE-J cells were cultured in OGD condition as described above. CLT concentrations were set at 2–50 µM. After culturing cells in 6-well culture plates, CellROX Orange Reagent (Invitrogen) was added directly to the cells in whole medium at a 1:500 dilution. Cells were incubated at 33 °C for 30 min, centrifuged once to remove medium and excess dye, and then resuspended in PBS. Cells were then analyzed with a Tali Image-Based Cytometer (Invitrogen) using the red fluorescent protein channel, collecting 9 fields per sample [29]. Untreated cells, which were also labeled with CellROX Orange Reagent, were used to determine baseline levels of oxidative activity and to set the fluorescent threshold for the Tali instrument. This threshold was set manually and confirmed visually. All cells with signals greater than the threshold value were counted by the Tali instrument as positive.

### Western blot analysis

RPE-J cells were cultured in OGD conditions by the same method described above. After treating cells with the indicated concentrations of CLT and exposing them to OGD conditions for 48 h, cells were washed with PBS, centrifuged, and then lysed using a ProteoJET Cell Lysis kit. Protein concentrations were determined using a bicinchoninic acid protein assay kit (Wako). Electrophoresis was performed using 4–15 % Tris–glycine gels. Proteins were transferred to PVDF membranes using a semidry transferring system (Biorad). The membranes were blocked with 5 % ECL blocking agent (GE Healthcare), incubated with primary antibodies against cleaved-caspase-3 (1:1000; Cell Signaling) and subsequently with the secondary antibody, horseradish peroxidase-linked IgG (1:10,000; Cell Signaling). After stripping the membranes of the antibodies for 10 min using reagents from a Western Re-Probe kit (Jacksun Biotech), the membrane was probed in a similar manner for β-tubulin (1:2000; Cell Signaling). Bands were visualized using an enhanced chemiluminescence system (ECL Plus, GE Healthcare). Band intensities were measured using Image J software.

### Device fabrication

The devices were fabricated as reported previously [13]. Briefly, the devices consist of a reservoir that can be loaded with a sustained release formulation of CLT and then sealed with a controlled release cover. PEGDM and TEGDM including 1 % 2-hydroxy-2-methylpropiophenone as a photoinitiator were used as device materials. The reservoir was prepared by pouring TEGDM prepolymer into a microfabricated polydimethylsiloxane mold followed by UV light (LC8, Hamamatsu Photonics) photopolymerization for 40 s at an intensity of 11.6 mW/cm^2^ [13]. The size of the reservoir was 2 mm × 2 mm wide × 1 mm high (drug-releasing surface area; 1.5 mm × 1.5 mm = 2.25 mm^2^) for the rat experiments and 4 mm × 4 mm × 1.5 mm (drug-releasing surface area; 3.5 mm × 3.5 mm = 12.25 mm^2^) for the CLT releasing in vitro assay. The amount of CLT released from the device for the rat experiments was small and was very difficult to detect by standard HPLC technique, so we decided to use a larger device for the in vitro release study. For the sustained release formulation, CLT was dissolved in a mixture of a PEGDM/TEGDM prepolymer mixture (40 %/60 %) having a CLT concentration of 250 mg/mL, and the mixture was poured into a reservoir and photopolymerized for 40 s. The loading volume was 1.2 μL and 12 μL for the rat experiments and in vitro release study, respectively. After loading the drugs, the reservoirs were covered by applying a prepolymer mixture with the required ratios of PEGDM and TEDGM to the reservoirs followed by UV exposure for 4 min. The volume of the PEGDM/TEGDM prepolymer mixture was 1 and 5 μL for the rat experiments and in vitro release study, respectively. PEGDM/TEGDM prepolymer mixture ratios of 0 %/100 %, 20 %/80 %, 40 %/60 % and 60 %/40 % were designated as P0, P20, P40, and P60, respectively. The placebo device was prepared using PBS as a drug formulation.

### In vitro release study

To demonstrate the controlled release of CLT, CLT was pelletized with P40 and loaded in the reservoirs, followed by sealing with P0, P20, P40 and P60 covers. Non-covered devices were prepared as controls. The devices were each incubated in 1.5 ml of PBS at 37 °C. 750 µl of PBS was collected at different intervals and mixed with 750 µl of acetonitrile and the amounts of CLT that had diffused out of the devices were measured using HPLC (Prominence, Shimadzu). A Shim-pack VP-ODS (Shimadzu) was used as a reversed-phase analytical column for CLT. The mobile phase of acetonitrile/10 mM sodium-phosphate buffer (pH 2.6) (4: 6, v/v) was delivered isocratically at 1 mL/min. The chromatograms were monitored at 215 nm. The devices were incubated in fresh PBS solution throughout the release study to ensure that the concentration of CLT would be below 20 % of its saturation value at all times. The results were expressed as amounts determined using a standard curve.

### Implantation

Male Sprague–Dawley rats (SLC) weighing 250–300 g were used in this study. All animals were handled in accordance with the Association for Research in Vision and Ophthalmology Statement for the Use of Animals in Ophthalmic and Vision Research after receiving approval from the Institutional Animal Care and Use Committee of the Tohoku University Environmental & Safety Committee (No. 2013MdA-218). The rats were anesthetized with ketamine hydrochloride (90 mg/kg) and xylazine hydrochloride (10 mg/kg). Their ocular surfaces were anesthetized with a topical instillation of 0.4 % oxybuprocaine hydrochloride. A paralimbal conjunctival incision was made 1 mm from the temporal limbus. The devices were placed onto the left eyes at the sclera. Three groups (each group was n = 3) were set to examine the effect of controlled CLT-release on retinal protection against light injury; first is for treatment with CLT-loaded devices and second is placebo devices (three per group) as control of drug effect, and third is non-light-exposed rats with non-treatment as control of light-induced injury effect.

### Light exposure

Seven days after device implantation, the unanesthetized rats were exposed to 8000 lux of white fluorescent light (Toshiba) for 24 h in automatically air-conditioned cages (22 °C) with a reflective interior (NK system). Before the light exposure, the rats were dark-adapted for 24 h and the pupils were dilated with 1 % cyclopentolate hydrochloride eye drops (Santen) 30 min before light exposure.

### Electroretinogram (ERG)

Four days after light exposure, flash ERGs were recorded as reported previously [30]. Briefly, after light exposure the rats were kept in the dark for 4 days until the flash ERGs were recorded (Purec, Mayo). The animals were anesthetized thirty minutes before the recording and the pupils were dilated with 1 % tropicamide and 2.5 % phenylephrine (Santen). Flash ERGs were recorded from the eyes of dark-adapted rats by placing a golden-ring electrode in contact (2.0 mm base curve, Mayo) with the cornea. An identical reference electrode was placed in the mouth, and a ground electrode was inserted subcutaneously near the tail. Single white-flash stimuli ranging from −3.5 to 0.5 log cd*s/m^2^ were used. All procedures were performed in dim red light, and the rats were kept warm using a heating pad (FHC-HPS RM25906, Muromachi Kikai) at 37 °C during the entire procedure. The amplitude of the a-wave was measured from the baseline to the maximum a-wave peak, and the b-wave was measured from the maximum a-wave peak to the maximum b-wave peak.

### Statistical analysis

Experimental data are presented as means ± standard deviations (SD). Statistical significance was calculated with Ekuseru-Toukei 2012 (Social Survey Research Information), using an unpaired *t* test for normally distributed isolated pairs, and the analysis of variance (ANOVA) with Tukey’s test for multiple comparisons. Differences were considered significant if *P* < 0.05 (*) or *P* < 0.01 (**).

## Results

### In vitro protective effects of CLT against oxidation-induced cell death

**Fig. 1:**
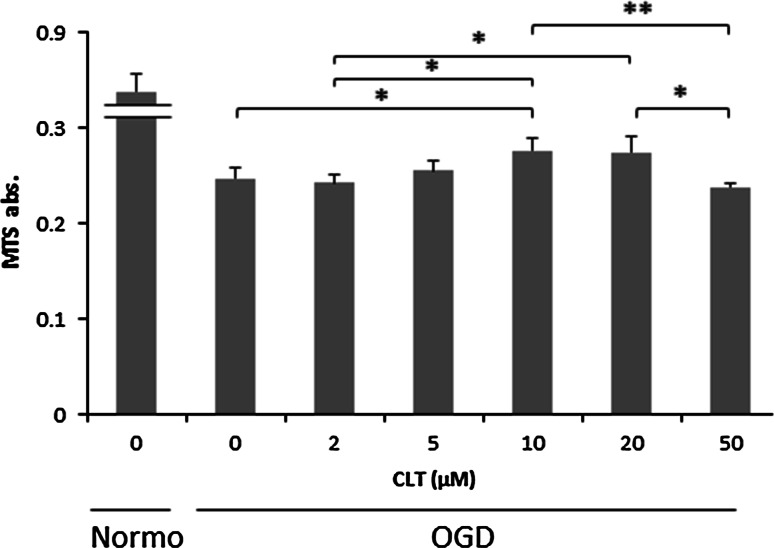
In vitro protective effects of administering different concentrations of CLT on the viability of RPE-J cell cultures under OGD stress conditions. Cell viability was measured using an MTS assay. Values are mean ± SD; n = 7. *P < 0.05., **P < 0.01. *Normo* normoxia

To investigate whether CLT could protect RPE-J cells against oxidative stress, and to clarify the effective doses of CLT, cells were pretreated with CLT and cultured under OGD conditions. An MTS assay was performed to evaluate cellular viability. The data confirmed that CLT-pretreated RPE-J cells showed significantly better cell survival under OGD conditions compared to non-pretreated cells and the increase in this viability was CLT-dose-dependent, i.e. administration of 10 µM showed the highest protection (Fig. 1). These data indicated that CLT might protect RPE-J cells from oxidation-induced cell death.

**Fig. 2:**
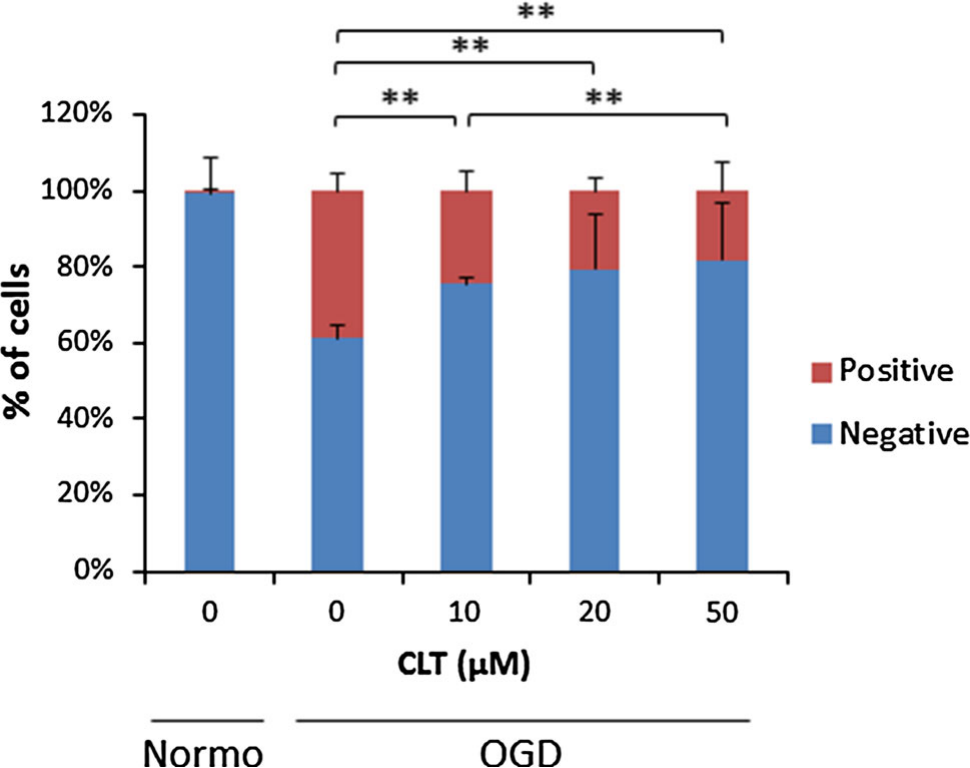
In vitro effects of CLT administration on ROS generation in RPE-J cell cultures under OGD stress conditions. ROS generation was assessed using a CellROX reagent and ROS-positive cells were measured by a Tali system (Invitrogen). Values are mean ± SD; n = 3. *P < 0.05, **P < 0.01. *Normo* normoxia (Color figure online)

To evaluate the preventive effects of CLT against ROS generation in RPE-J cells, these cells were pretreated with predetermined concentrations of CLT and exposed to OGD conditions. Results are shown as the proportions of ROS positive cells against negative ones. The data indicated that intracellular ROS was increased in the OGD condition as up to 40 % comparing to the normoxia group, which was, however significantly reduced by administration of 10, 20 and 50 μM CLT treatment consequently (Fig. 2). These data indicated that inhibition of ROS generation by CLT application might protect RPE-J cells from oxidation-induced apoptosis.

**Fig. 3:**
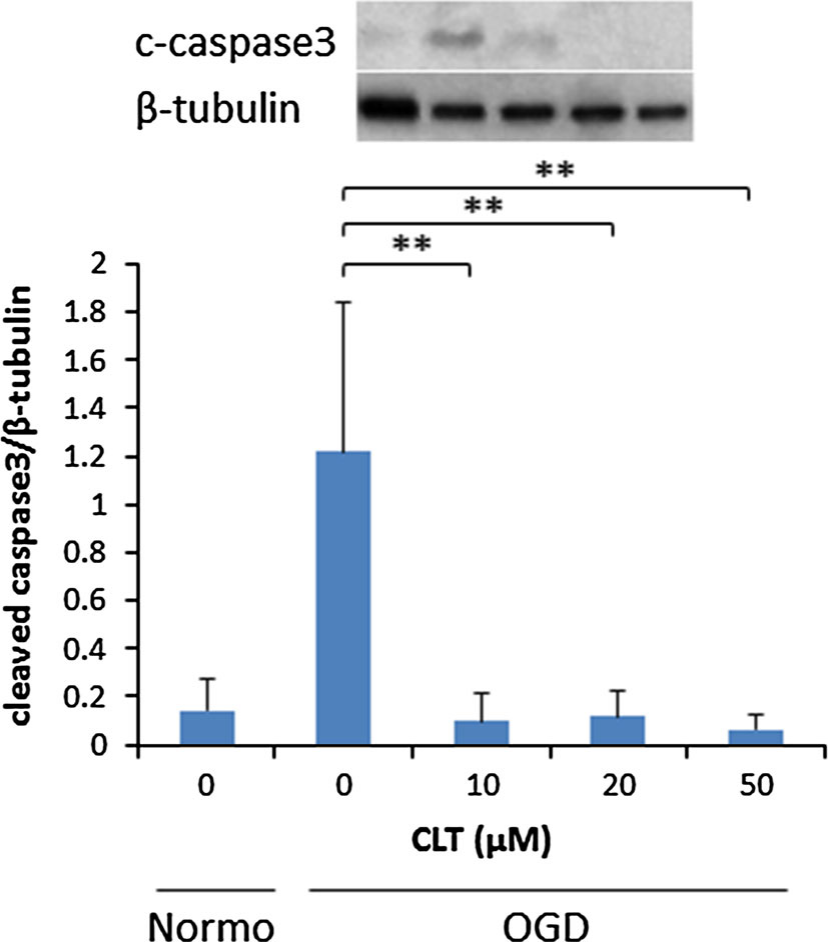
Western blotting of cleaved caspase-3 in CLT-pretreated RPE-J cells cultured under OGD conditions. The band intensities relative to beta-tubulin are shown in the bar graph. Values are mean ± SD; n = 4. *P < 0.05, **P < 0.01. *Normo* normoxia

Whether the cytoprotective effect of CLT on RPE-J cells is related to caspase-3 activity was assessed. RPE-J cells cultured under OGD-induced oxidative stress conditions exhibited elevated cleaved caspase-3 protein levels. The increased protein level was inhibited by pre-treatment with CLT (Fig. 3).

### Device fabrication and in vitro release study

**Fig. 4:**
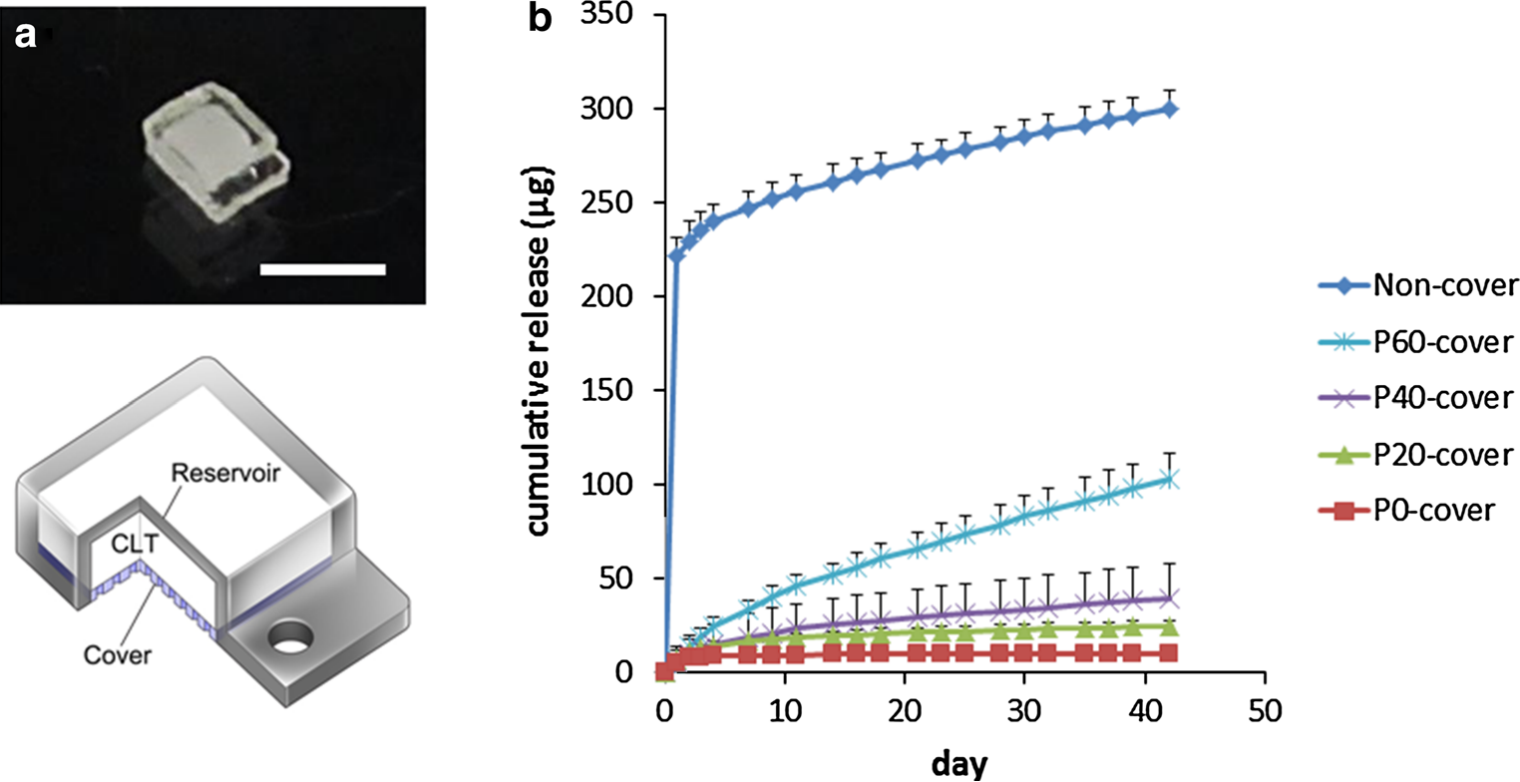
Transscleral intraocular drug delivery system and in vitro release of CLT from drug delivery devices. **a** Image shows a device that consists of the CLT pelletized with PEGDM/TEGDM, a reservoir made of TEGDM, and a controlled release cover made of a PEGDM/TEGDM mixture. **b** Release profiles of the drug-delivery device consisting of CLT pelletized with P40 and various types of cover (P0, P20, P40, and P60) or non-cover. Values are mean ± SD; n = 4

The device consists of a separately fabricated reservoir, a CLT formulation, and a controlled release cover (Fig. 4a). The CLT/P40 mixture (250 mg/mL CLT) was loaded into the reservoirs and the reservoirs were covered by different formulations of PEGDM/TEGDM polymer (P60, P40, P20, and P0). A prominent initial increase following a constant release was observed in the non-covered device (Fig. 4b). The constant release after a burst-like release is related to the sustained release formulation made of PEGDM/TEGDM. In the covered devices, a minor increase was observed initially, after which the release rate was almost constant and was dependent on the PEGDM/TEGDM ratio of the cover (Fig. 4b). The release rates estimated from the gradient curves for P60-, P40-, P20- and P0-covered devices were 1.86, 0.53, 0.20, and 0.01 μg/day, respectively. These results demonstrate the ability to control the release rate from a device by changing the ratio of PEGDM/TEGDM.

### In vivo protective effects of CLT against light-induced retinal damage in rats

**Fig. 5:**
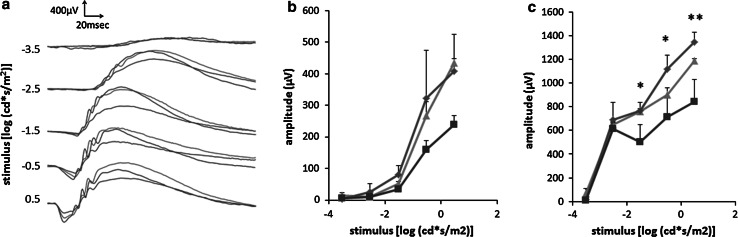
Effect of sustained CLT administration using this device on retinal protection. **a** Representative ERG spectra (*blue diamonds* CLT-loaded device-treated rats, *red squares* Placebo treated rats, *green triangles* non-light-exposed rats with non-treatment) and ERG amplitudes of **b** a- and **c** b-waves in the P40-covered device-treated group. Values are mean ± SD; n = 3. *P < 0.05, **P < 0.01 (Color figure online)

To determine the influence of sustained CLT administration by our controlled release device against light-induced retinal damage in rats, ERG amplitudes of a- and b-waves were measured (Fig. 5a). Light exposure was performed 7 days after implantation when the acute inflammatory phase caused by device implantation might disappear. In the absence of CLT treatment, the ERG amplitudes in the light-exposed rats decreased to approximately 50 % of those of non-exposed rats. The amplitudes of a- and b-waves recorded from rats treated with CLT-loaded devices with P40-covers were higher than those that received a placebo device treatment and comparable to those of non-light-exposed rats (Fig. 5b, c), and a statistically significant difference was observed in the amplitudes of b-waves (Fig. 5c).

## Discussion

As CLT is administered via a transscleral route, the RPE should be the first retinal tissue exposed to released CLT. Our previous study showed that transsclerally delivered compounds may accumulate around the RPE [13]. The cumulative oxidative damage to RPE cells resulting from ROS generation is reported to be one of the pathological conditions in AMD [7]. Therefore, we first investigated the pharmacological action of CLT against RPE cells. CLT is a member of a large and structurally diverse group of azole fungicides that act by inhibiting cytochrome P450-dependent sterol 14-demethylases and hence are capable of blocking sterol synthesis [31]. CLT has been shown to have diverse effects on cellular metabolism and signaling pathways, particularly Ca^2+^-dependent processes [17, 32]. Moreover, CLT has a free radical scavenger effect, which has been observed in several cell types [21]. It is possible that the reduction in caspase-3 activity and ROS generation by CLT is causally associated with the inhibition of oxidative stress-induced cell death. Many researchers have investigated the efficacy of antioxidants such as ascorbate [33, 34], dimethylthiourea [35], thioredoxin [36], phenyl-*N*-tertbutylnitrone [37, 38], and retinal defensive agents including superoxide dismutase against light-induced retinal damage [39]. However, many factors play a part in oxidative stress and ROS generation [40]. The mechanisms described above may be only partially responsible for the protective effect of CLT via its inhibition of oxidation-induced cell death. The other possible mechanisms and pathways have yet to be elucidated.

The establishment of safe and effective methods for administering drugs to the retina has been an obstacle to developing effective new therapies for ocular disorders. In recent years, various drug delivery systems have been developed to circumvent the side effects of conventional methods including intravitreal injections, and to improve the ocular bioavailability of eye drop-administered drugs [41]. This study demonstrated that CLT can be applied via a polymeric drug delivery system [13] and can be released in a controlled manner. The ability to control the drug release is based on the swelling of the PEGDM/TEGDM polymer [13]. A polymer made of short chains of TEGDM is likely to be compact, allowing practically no penetration of drugs. In fact, as pure TEGDM (P0) is almost impermeable to CLT, it enables unidirectional release to the sclera through the cover with negligible amounts through the TEGDM reservoir. In the other hand, long chains of PEGDM result in a greater tendency to swell and an open polymer network, facilitating permeation of CLT through the PEGDM/TEGDM cover. The drug loading of the devices used in the in vitro study was 3 mg (250 mg/mL × 12 μL). Therefore, even in the case of the P60-covered device with a release rate of 1.86 μg/day, a sustained release for over 4 years may be possible. Consequently, CLT could be released in a controlled release manner by a PEGDM/TEGDM system.

Our preliminary implantation study in rats shows that a CLT-loaded device implanted on the sclera could protect retinal function against light injury, based on measurements of ERG amplitudes. In the rat experiments, the amounts released would be less than the release profiles shown in Fig. 4b, because we used a smaller device for the in vivo study than used in the in vitro release assay. The larger device used in the in vitro study had 5.44 times larger drug-releasing surface area (12.25 mm^2^ vs. 2.25 mm^2^) and 3.42 times faster release rate than that of the device used in rats [42]. Therefore, the actual release rate and the duration of release in the rat experiments might be from one-third to one-fifth of the in vitro release profiles shown in Fig. 4b. As the in vitro cell culture results showed a protective effect at 10 μM (0.34 μg/mL)-CLT administration, the P40-covered device for rats, which may have a release rate of between 0.10–0.17 μg/day (one-fifth to one-third of 0.53 μg/day) was used in the in vivo study. Significant attenuation of b-wave amplitude reductions by CLT administration might indicate a potent protection of photoreceptors.

A limitation of this study is the lack of data on the pharmacokinetics of CLT in the eye, because the rat eyeballs were too small to measure the level of CLT in the retina using HPLC. Although we have analyzed the transport of fluorescents as model drugs into the eye from the device [13], the quantification of the CLT distribution in the eye requires using larger animals. Additionally, the duration of the effect and the appearance of side effects after long-term implantation remain to be determined. To further support the CLT-mediated retinal protection, we are planning on carrying out a classical histological analysis of retinal sections and protein/gene expression assays in the future. Furthermore, there is a growing awareness of the limitations of animal research and its inability to make reliable predictions for human clinical trials [43]. The problem with most of today’s animal studies is that they rarely predict exactly what will happen in the clinic, because the doses, formulations and schedules of medication differ from those given to the animals [44]. Similarly our drug delivery system also encounters with limited ability of animal models to mimic exactly the extremely complex process of human physiology and also the difference of the eye size between humans, which affects the effective doses of the applied drug and the size and the implantation method of the delivery device. Therefore, animal models are another complication in translating the results of the in vivo findings to human models.

## Conclusion

In summary, the present study reveals that CLT can protect RPE-J cells against OGD-induced oxidative stress via the suppression of ROS generation and caspase-3 activity. Furthermore, a sustained CLT release system was established and its potent retinal protective effects demonstrated using a light-induced retinal damage animal model. We conclude that CLT may be a promising candidate for the treatment of visual impairment caused by AMD.

## Abbreviations

CLT: Clotrimazole
RPE-J: Retinal pigment epithelium cell line
OGD: Oxygen/glucose deprivation
